# The impact of residual infections on *Anopheles*-transmitted *Wuchereria bancrofti* after multiple rounds of mass drug administration

**DOI:** 10.1186/s13071-015-1091-z

**Published:** 2015-09-24

**Authors:** Dziedzom K. de Souza, Rashid Ansumana, Santigie Sessay, Abu Conteh, Benjamin Koudou, Maria P. Rebollo, Joseph Koroma, Daniel A. Boakye, Moses J. Bockarie

**Affiliations:** Noguchi Memorial Institute for Medical Research, University of Ghana, Legon, Ghana; Centre for Neglected Tropical Diseases, Liverpool School of Tropical Medicine, Liverpool, UK; Mercy Hospital Research Laboratory, Bo, Sierra Leone; Ministry of Health and Sanitation, Freetown, Sierra Leone; Task Force for Global health, Atlanta, USA

**Keywords:** Lymphatic filariasis, *Wuchereria bancrofti*, Residual transmission, Hotspots, Sierra Leone

## Abstract

**Background:**

Many countries have made significant progress in the implementation of World Health Organization recommended preventive chemotherapy strategy, to eliminate lymphatic filariasis (LF). However, pertinent challenges such as the existence of areas of residual infections in disease endemic districts pose potential threats to the achievements made. Thus, this study was undertaken to assess the importance of these areas in implementation units (districts) where microfilaria (MF) positive individuals could not be found during the mid-term assessment after three rounds of mass drug administration.

**Methods:**

This study was undertaken in Bo and Pujehun, two LF endemic districts of Sierra Leone, with baseline MF prevalence of 2 % and 0 % respectively in sentinel sites for monitoring impact of the national programme. Study communities in the districts were purposefully selected and an assessment of LF infection prevalence was conducted together with entomological investigations undertaken to determine the existence of areas with residual MF that could enable transmission by local vectors. The transmission Assessment Survey (TAS) protocol described by WHO was applied in the two districts to determine infection of LF in 6–7 year old children who were born before MDA against LF started.

**Results:**

The results indicated the presence of MF infected children in Pujehun district. *An. gambiae* collected in the district were also positive for *W. bancrofti*, even though the prevalence of infection was below the threshold associated with active transmission.

**Conclusions:**

Residual infection was detected after three rounds of MDA in Pujehun – a district of 0 % Mf prevalence at the sentinel site. Nevertheless, our results showed that the transmission was contained in a small area. With the scale up of vector control in *Anopheles* transmission zones, some areas of residual infection may not pose a serious threat for the resurgence of LF if the prevalence of infections observed during TAS are below the threshold required for active transmission of the parasite. However, robust surveillance strategies capable of detecting residual infections must be implemented, together with entomological assessments to determine if ongoing vector control activities, biting rates and infection rates of the vectors can support the transmission of the disease. Furthermore, in areas where mid-term assessments reveal MF prevalence below 1 % or 2 % antigen level, in *Anopheles* transmission areas with active and effective malaria vector control efforts, the minimum 5 rounds of MDA may not be required before implementing TAS. Thus, we propose a modification of the WHO recommendation for the timing of sentinel and spot-check site assessments in national programs.

## Background

The Global Program to Eliminate Lymphatic Filariasis (GPELF) targets the elimination of LF as a public health problem by the year 2020, through mass drug administration (MDA) in endemic implementation units (IU), with the aim of interrupting transmission and stopping the spread of infection in all endemic areas. [1]. While many countries have made significant progress in reducing transmission intensity and incidence of infection through community-wide treatments, there remain significant programmatic challenges to interrupting parasite transmission. These include effective implementation of the preventive chemotherapy strategy in urban settings, [2, 3] and the existence of areas of residual infection [4–6] that may precipitate the spread of infection after the conditions for stopping MDA have been met [7, 8].

Implementing MDA is a critical challenge for the GPELF, especially in countries affected by conflict. Among the four LF endemic countries (Sierra Leone, Liberia, Guinea and Cote d’Ivoire) recently affected by conflict in West Africa, only Sierra Leone was implementing MDA (with Ivermectin and Albendazole) in 2011. All 14 districts in Sierra Leone were endemic for LF antigen before MDA started in 2008 [9, 10]. Nevertheless, after three rounds of treatment (2008–2010), a midterm progress evaluation following WHO guidelines revealed that the microfilaria prevalence in people five years and older was reduced to 0 % in five districts [9]. The other nine districts had microfilaria (MF) prevalence below 1 % in sentinel sites with the exception of one district. The overall average MF prevalence, before and after the three MDAs, were 2.4 % and 0.3 % respectively [9].

LF and onchocerciasis are co-endemic in 12 of the 14 districts in Sierra Leone. Prior to the initiation of MDA for LF in Sierra Leone in 2007, many people in the implementation units co-endemic for both diseases had received more than 5 rounds of treatment with Ivermectin through the community directed intervention (CDI) implemented by the African Programme for Onchocerciasis Control (APOC) [11]. Treatment for onchocerciasis and, scaling up of bed net distribution in Sierra Leone [12, 13] may have impacted LF prevalence because Ivermectin alone is also effective against LF [14–16], and treated bed nets dramatically reduce exposure to mosquito bites [17]. Furthermore, in Sierra Leone as in other countries in West Africa, LF is transmitted solely by the malaria carrying *Anopheles* mosquitoes [18]. *Anopheles*-transmitted LF is highly focal [19–21] and synchronous with intense malaria transmission [21, 22]. Malaria control efforts targeting *Anopheles* mosquitoes therefore have the potential to significantly impact on LF transmission in West Africa, as was possibly the case in the interruption of LF transmission in Togo [23].

WHO recommends that a Transmission Assessment Survey (TAS) to determine when to stop MDA be carried out when an implementation unit (District) has completed five effective rounds of annual MDA and the prevalence of MF is less than 1 % in all sentinel and spot check sites in the districts [24]. TAS is based on antigenemia prevalence (in children) that may persist after transmission has been interrupted. Interpretation of these endpoints is also confounded by the size of the evaluation unit, focality of the disease and movement of infected individuals from endemic areas to non-endemic areas. Despite the fact that Sierra Leone had only implemented three effective rounds of MDA for LF, this study was undertaken to investigate the significance of residual infections for the outcome of TAS in areas of *Anopheles*-transmitted LF previously treated with Ivermectin, and with active vector control activities.

## Methods

### Ethics statement

Approval for the study was obtained from the ethics review committee of the Liverpool School of Tropical Medicine and the Ministry of Health – Sierra Leone. Prior to conducting the survey and obtaining informed consent, repeated community meetings were held in all of the villages to communicate the purposes of the study and answer questions at the individual and community level. Informed consent to participate in the study was obtained from all individuals 18 years or older. Consent was obtained from a parent or guardian of younger individuals. Informed consent was also received from mosquito collectors, 18 years and above, after which they were trained in safe and scientifically reliable mosquito collection. Consent was also sought from the head of the households where mosquito sampling was carried out.

For the Transmission Assessment Surveys (TAS), the communities where the schools were located were informed of the purpose of the study, in their local language. Due to low literacy rates, informed oral consent was obtained from the community leaders, as well as parents and guardians of each child participating in the study. The names of consenting parents and their children were recorded, and only the principal investigators of the study had access to this information. The data was analysed and reported, to exclude any directly identifiable information, in order to maintain the anonymity of the parents and children.

### Study sites

This study was conducted in the Pujehun and Bo Districts of Sierra Leone. Bo town is the second largest city and an important mining centre in Sierra Leone, whereas Pujehun is a less populated, semi-urban area. These districts are located in the rainforest area in the Southern Province, with farming as an important socio-economic activity. Baseline MF (2007–8) and midterm (2011) surveys failed to identify any MF positive individual in one of the two districts studied (Pujehun), which was maintained in this district, and the number of MF positive individuals in the second district (Bo) reduced from 2.0 % to zero after three rounds of MDA [9, 10]. Communities in the districts were visited to assess their sizes and distances from the district capitals, in order to plan the entomological investigations. The number of households was determined from the community data. Based on this information study communities were purposefully selected to maximize the collection of mosquitoes, taking into consideration logistic demands. Six communities in the Pujehun Districts and four communities in the Bo District were selected for the study (Table 1). The coordinates of the communities and distance from the district capital were recorded using a Garmin Handheld GPS. The map of the study communities was drawn in ArcGIS version 10 (ESRI).

**Table 1:** Surveillance for LF in study sites in Pujehun and Bo Districts. *Schools in these communities were part of the TAS survey

District	Site	Females	Males	Total Tested	ICT Positive (%)
Pujehun	Sahn Malem	124	226	350	0 (0.0)
	Karlu*	188	162	350	1 (0.3)
	Gbondapi	192	158	350	3 (0.9)
	Sumbuya Bessima	31	32	63	0 (0.0)
	Kondorwahun	65	92	157	0 (0.0)
	Vaama*	144	127	271	1 (0.4)
	Total	744	797	1542	5 (0.3)
Bo	Njala Komboya	164	186	350	3 (0.9)
	Nyandeyama	144	183	327	6 (1.8)
	Nengbema*	153	197	350	0 (0.0)
	Mendewa	126	160	286	0 (0.0)
	Total	587	726	1313	9 (0.7)

### Sample collection

Assessment for LF prevalence was conducted in the study communities using the Binax Now ICT card. Altogether, 1542 individuals were surveyed in the Pujehun District while 1313 individuals were surveyed in the Bo District. Samples were collected from individuals aged between 6–65 years of age.

Entomological surveys were also undertaken to determine the existence of active transmission in study areas, and also study the importance of local transmission. Thus, mosquitoes were collected using the Pyrethrum Spray method, Exit Traps and Human Landing Collections, and processed as previously described [25]. Briefly, DNA was extracted from pooled mosquitoes using the Qiagen DNeasy tissue kit (Qiagen CA) extraction method. This was followed by PCR to detect *W. bancrofti* DNA using the method of Ramzy and colleagues [26]. A positive and negative control was included in all reactions and samples testing positive for *W. bancrofti* were confirmed using a second PCR. Positive samples were also confirmed using the slightly modified loop-mediated isothermal amplification (LAMP) method for detecting *W. bancrofti* DNA [27]. In each household surveyed for mosquitoes, we collected information on the number of people living in the house and the number of people who used ITN the night before the collection. From this information ITN usage rates were determined for each community.

While this study was not meant to undertake Transmission Assessment Surveys (TAS) [24], the TAS protocol was applied in the two districts because it is a statistically strong method to determine infection prevalence of LF in children as an indicator of active transmission in the district. TAS for LF is undertaken to determine whether infection has been reduced to levels below which transmission cannot be sustained, allowing for a decision to stop MDA. As such, children aged 6–7 years were sampled from various schools selected using the TAS protocol, and tested for LF antigen and microfilaria. Some schools in the LF assessment sites formed part of the schools selected using the TAS protocol.

### Statistical analysis

Infection in the vector population was calculated using the Poolscreen v2.0 [28] to determine the maximum likelihood of infection together with the associated 95 % CIs. From the ICT and TAS survey, the prevalence (%) of antigenemia and microfilaremia was calculated as the number of positive people divided by the number of people examined.

## Results

The cross sectional surveys revealed that out of the six communities surveyed in Pujehun district, three were positive for *W. bancrofti* infection using ICT cards (Table 1). In Bo, two communities out of four were positive for antigen. The total antigen prevalence in the districts was 0.3 % (5/1542) and 0.7 % (9/1313) in the Pujehun and Bo districts respectively.

Following the TAS protocol, ten antigen and four MF positive children were identified in the Pujehun district (Table 2) [25], while only three antigen positive children were detected in Bo. No MF positive children were detected in Bo. The antigen levels following the school cluster surveys were 0.67 % and 0.16 % in Pujehun and Bo respectively. Despite the antigen levels being below the recommended prevalence for stopping MDA in the districts, antigen levels were ≥ 2 % in some of the school clusters used in the TAS protocol (Table 3).

**Table 2:** TAS summary results for school children in Pujehun and Bo Districts [25]

District	No. of Schools	No. of Children Surveyed	Males (%)	Females (%)	No. MF Positive	No. Ag. Positive (%)	Critical Cut-off Value for Ag positives
Pujehun	31	1503	659 (43.8 %)	844 (56.2 %)	4	10 (0.67 %)	18
Bo	30	1564	682 (43.6 %)	882 (56.4 %)	0	3 (0.16 %)	18

**Table 3:** School clusters positive during the TAS survey

	Names of schools	Town/Village	Total Tested	No. of Ag. Positives (%)	No. of MF Positives (%)
Pujehun	Roman Catholic school	Potoru-Zimmi Rd	50	3 (6.0)	2 (4.0)
	United Muslim Association	Tongay/Pujehun	42	3 (7.1 %)	0
	SLC Primary School	Boma	50	1 (2.0)	1 (2.0)
	SLC Primary School	Karlu*	50	1 (2.0)	1 (2.0)
	SLC Primary School	Mano Gbojeima	50	1 (2.0)	0
	Roman Catholic school	Zimmi Makpele	50	1 (2.0)	0
Bo	S.D.A. Samamie	Bo	59	1 (1.7)	0
	UMC Jembeh	Jembeh	52	1 (1.9)	0
	UMC Primary School	Benduma	49	1 (2.0)	0

**Fig. 1:**
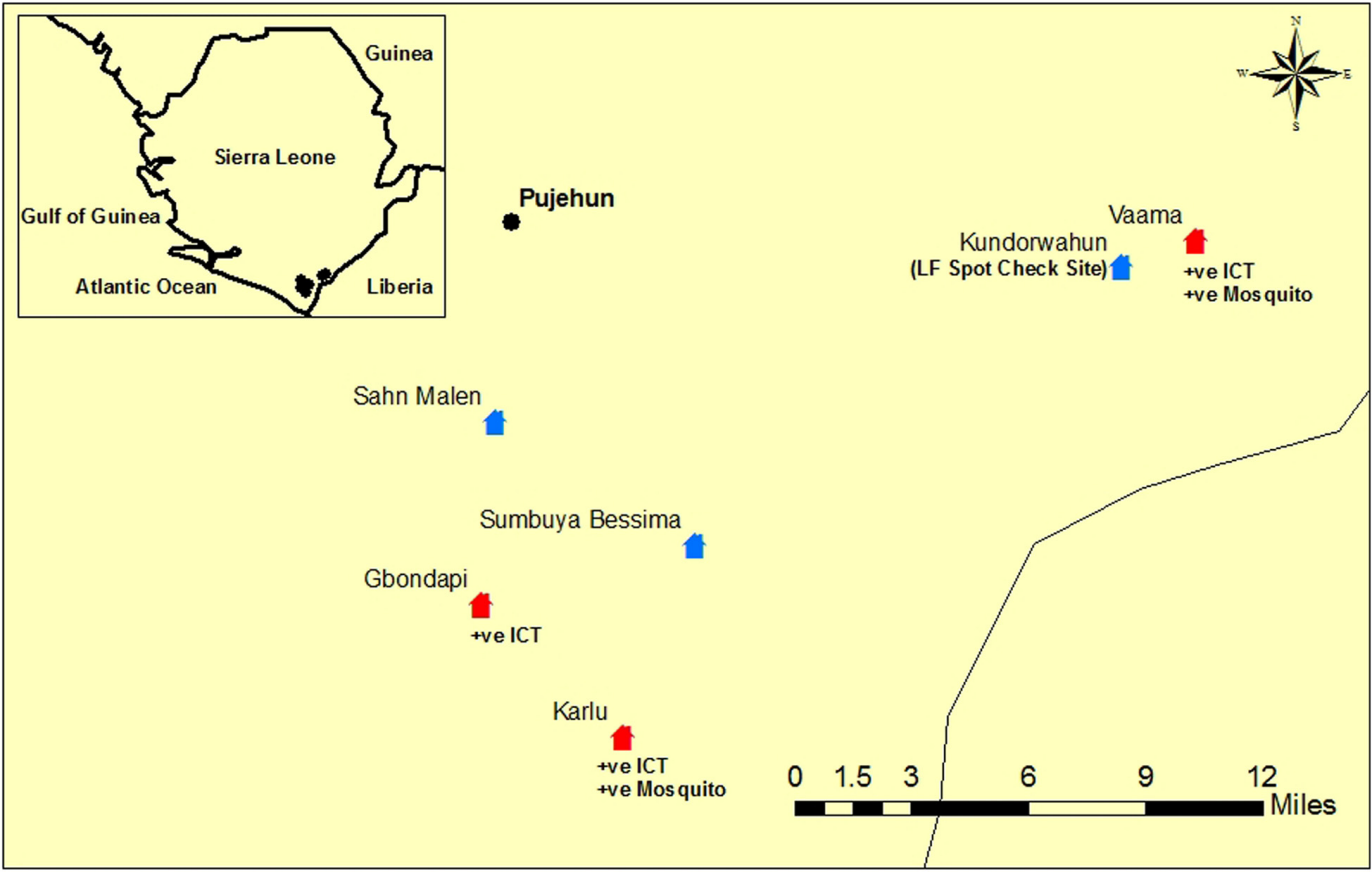
Map of survey sites in Pujehun District

The entomological surveys revealed that low numbers of *An. gambiae* were caught in the study villages and processed for *W. bancrofti* infection (Table 4). In Pujehun, a total of 259 *An. gambiae* mosquitoes were processed for *W. bancrofti* infection in 21 pools (pool range 3–20). Despite the low numbers of mosquitoes collected and processed, molecular xenomonitoring revealed two pools positive for *W. bancrofti* DNA, with a Maximum Likelihood Infection (MLI) estimate of 0.79 % (Table 4), in communities where antigen positive individuals were identified (Fig. 1). In Bo, 791 mosquitoes were collected and no positive mosquitoes were detected. The ITN usage in the districts was also estimated to be 66.1 % (193/292) in Pujehun and 49.3 % (621/1260) in Bo.

**Table 4:** Xenomonitoring results from Pujehun and Bo Districts

Districts	Sites	ITN Usage (%)	No. of mosquitoes	No. of pools	Pools positive (MLI %)	95 % CI
Pujehun	Sahn Malen	30/59 (50.8)	65	5	0	-
	Karlu*	22/37 (59.5)	75	5	1 (1.42)	0.044 - 7.1
	Gbondapi	31/46 (67.4)	18	2	0	-
	Sumbuya Bessima	35/73 (47.9)	14	2	0	-
	Kundorwahun	42/44 (95.5)	36	2	0	-
	Vaama	33/33 (100.0)	51	5	1 (2.04)	0.064 - 10.1
	Total	193/292 (66.1)	259	21	2 (0.79)	0.094 - 2.76
Bo	Njala Komboya	53/146 (36.3)	135	8	0	-
	Nyandeyama	213/343 (62.1)	492	26	0	-
	Nengbema	174/232 (75.0)	80	5	0	-
	Mendewa	181/539 (33.6)	84	6	0	-
	Total	621/1260 (49.3)	791	45	0	-

## Discussion

The results of the antigen prevalence survey revealed that levels were below the thresholds that signify sustainable transmission [25]. However, it is worth mentioning that even though the antigen levels were below the recommended levels for the entire districts, antigen levels were ≥ 2 % in some of the school clusters used in the TAS protocol, with similar observations made in Sri Lanka and Zanzibar [5, 29]. During the mapping surveys in 2008, Pujehun was one of the districts with low endemicity of antigenemia (4 %), with no microfilaria detected [10]. Nonetheless, our study revealed the existence of areas with residual transmission in Pujehun district with evidence of active but highly focal transmission of LF through the detection of MF in children and uptake of MF by LF vectors. While areas of residual transmission may be termed as hotspots, it is important to clearly establish what can be considered a hotspot in LF transmission. We considered a hotspot to be: 1. an area where MF carriers persisted after 3 or more rounds of MDA when the sentinel site prevalence is less than 1 %; 2. school clusters with antigen prevalence of > 2 % in 6–7 year olds, following TAS. The existence of areas of residual transmission in Pujehun District (Fig. 1) illustrates the focality of *Anopheles*-transmitted LF and challenges faced in selecting high risk areas for sentinel site and spot check surveys, and the need for adopting more statistically robust sampling strategies and reviewing the size of the evaluation unit for TAS [4].

The low numbers of mosquitoes collected in the study areas is probably the result of the high ITN usage in the study areas (66.1 % and 49.3 % in Pujehun and Bo respectively). There has been an increased use of insecticide treated nets through mass ITN distribution campaigns in Sierra Leone and in the Pujehun and Bo Districts [12, 13]. By 2010, 67.2 % LLIN usage was reported in the study areas [13]. Prior to this, the use of ITN has never been tried in forest zones, and the introduction of ITN in Bo district followed earlier studies to evaluate the Anopheline ecology and behaviour, to understand the role of the vectors in malaria epidemiology and formulate appropriate strategies for the area [30–33].

The detection of positive mosquitoes in areas positive for antigen in humans indicates possible on-going transmission, and similar results have been obtained in the American Samoa [34]. These results support the evidence that molecular xenomonitoring can be an effective tool in post-MDA surveillance [5, 34]. While there is currently no existing target threshold for monitoring parasite DNA prevalence in Anopheline vectors [5, 35], 0.25 % has been suggested as the maximum infection prevalence expected to sustain transmission by *Culex* species [5]. Studies are required to determine cut-off threshold for *Anopheles* mosquitoes. Further, given that *Culex* mosquitoes are more efficient LF vectors than *Anopheles*, we advocate the establishment for different cut-offs for these species by TAS, as the current algorithm for choosing TAS design is similar in areas where LF is transmitted by *Anopheles* and *Culex* species [24].

In Sierra Leone, as in the other countries in West Africa, lymphatic filariasis is transmitted by the malaria carrying *Anopheles* mosquitoes and *Culex* species play little or no role in the transmission of the disease [18]. Very early studies elsewhere have shown that where LF transmission by *Anopheles* mosquitoes was interrupted through vector control alone, transmission never resumed. The control of vectors through house-spraying with residual insecticides resulted in the sustained interruption of LF by the *Anopheles punctulatus* group in Solomon Islands [36] and parts of Papua New Guinea [37], and cases of LF resurgence only detected in countries where *Culex* mosquitoes were the vectors, including Zanzibar in United Republic of Tanzania [7, 8, 29]. As suggested by Webber, vector biting rates of Anopheline mosquitoes less than 0.66 bites/man/h are unlikely to sustain the transmission of LF [36], and malaria control efforts targeting the *Anopheles* mosquito therefore have the potential to impact LF transmission in Africa [23, 38], except in areas where some species of Anopheline mosquitoes have the potential to exhibit the phenomenon of limitation [39, 40]. As such, what is the significance of areas of residual transmission on the elimination of LF? Studies elsewhere, including Mali in West Africa, have shown that the existence of areas of possible transmission did not result in resurgence of the disease [36, 37, 41]. Before China was certified free of LF in 2007, studies had shown that despite the presence of residual MF prevalence in the population, transmission was considered to have been interrupted [42, 43]. This does lead us to operate on the hypothesis that the threshold for active transmission of LF in areas where *Anopheles* mosquitoes exhibit facilitation is higher compared to areas where *Culex* mosquitoes are principal vectors. As such, these observations bring into question the importance of areas with residual infections on the elimination of LF.

## Conclusion

**Fig. 2:**
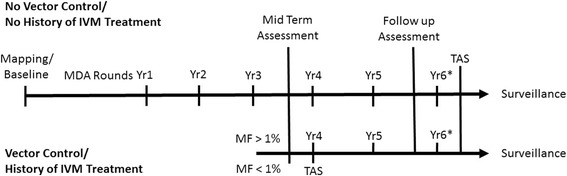
Modification of the WHO recommendation [1] for timing of sentinel and spot-check site assessments in national programmes. * Likely, but not necessary, to be conducted regardless of assessment results

From this study and other reports from elsewhere, we conclude that the existence of areas of residual transmission will not necessarily lead to the spread of *Anopheles* transmitted LF infection, where the vectors exhibit facilitation. What should be emphasized is the value of xenomonitoring in determining if ongoing vector control activities, biting rates and infection rates of the vectors can support the transmission of the disease. Additional control strategies may then be implemented based on the evidence obtained from the xenomonitoring surveys in these areas. Furthermore, it may not be necessary to complete the minimum 5 rounds of MDA before implementing TAS, when mid-term assessments reveal MF prevalence below 1 % or 2 % antigen level, in *Anopheles* transmission areas with active and effective malaria vector control efforts. Implementing 2 additional rounds of MDA before TAS in these areas will require significant resources that can better be directed to other areas with more pressing needs. Thus we propose a modification (Fig. 2) of the WHO recommendation for the timing of sentinel and spot-check site assessments in national programs [1], depending on whether LF endemic areas have a history of Ivermectin treatment and/or implement vector control strategies which may differ in various countries, through ITN/LLIN distribution or Indoor Residual Spray.
